# LB2305. Long Term Benefits from Early Antiretroviral Therapy Initiation in HIV Infection: Findings from the Extended Follow-up of the START Trial

**DOI:** 10.1093/ofid/ofac492.1895

**Published:** 2022-12-15

**Authors:** Abdel G Babiker, Jens Lundgren, Shweta Sharma, Birgit Grund, Andrew Phillips, Virginia L Kan, Gail Matthews, Karin L Klingman, James D Neaton

**Affiliations:** University College London, London, England, United Kingdom; Rigshospitalet, University of Copenhagen, Copenhagen, Hovedstaden, Denmark; University of Minnesota, Minneapolis, Minnesota; University of Minnesota, Minneapolis, Minnesota; UCL, London, England, United Kingdom; VA Medical Center, Washington, District of Columbia; Kirby Institute, UNSW, Sydney, New South Wales, Australia; Medical Science and Computing, Silver Spring, Maryland; University of Minnesota, Minneapolis, Minnesota

## Abstract

**Background:**

Deferral of antiretroviral therapy (ART) initiation until CD4 count < 350 cells/μL in people with CD4 > 500 increases risk of AIDS and serious non-AIDS (SNA) conditions. Evidence from randomized trials is lacking to assess the extent to which ongoing excess risk due to having deferred initiation is reduced once ART is initiated and viral load suppressed.

**Methods:**

START randomized 4684 ART-naïve HIV+ adults with CD4 > 500 cells/μL to start ART immediately (IMM, n=2325) or defer ART until CD4 < 350 cells/μL or AIDS developed (DEF, n= 2359). In 2015 the study demonstrated a 57% reduction in the risk of the primary endpoint (AIDS, SNA or death) for IMM ART; all assigned to DEF were recommended to start ART. Follow up continued to 31Dec2021. To determine whether the benefit of IMM compared to DEF was maintained, increased, or reduced, Cox models were used to estimate and compare hazard ratios (HR, IMM:DEF), for the primary endpoint and its components from randomization through 31Dec2015 (Pre 2016) with the period 1Jan2016 through 31Dec2021 (Post 2016). HRs for all follow-up, randomization through 31Dec2021, were also estimated.

**Results:**

Pre 2016, median CD4 was 648 cells/μL in IMM and 460 cells/μL in DEF at ART initiation, a difference of 188 cells/μL (95% CI: 180-197); at the end of 2015, 98.9% of IMM and 84.8% of DEF were prescribed ART. The percentage of follow-up time spent on ART pre 2016 was 94.5% for IMM and 35.5% for DEF; and the average difference in CD4 count was 199 cells/μL. This contrasts with post 2016 during which the percentage of follow-up time on ART was 99.5% for IMM and 95.6% for DEF; and the average difference in CD4 was 155 cells/mL (Figure 1).

During both time periods, initiation of ART led to rapid declines in HIV RNA to ≤ 200 copies/mL. For the primary endpoint and each component, the benefit of IMM compared to DEF in Post 2016 was reduced compared to Pre 2016 (Figure 2).

Overall follow-up, from randomization to 2021 (median 9.3 years), the HR for the primary endpoint was 0.61 (95%CI: 0.49, 0.76; p< 0.001).

**Conclusion:**

Among people with CD4 > 500, the excess risk of AIDS and SNA conditions associated with delaying ART initiation is substantially diminished soon after ART is initiated, but persistent excess risk remains. The results reinforce the importance of early diagnosis of HIV and prompt initiation of ART.

**Disclosures:**

**Gail Matthews, MBChB, PhD**, Abbvie Inc: Grant/Research Support|Astra Zeneca: Advisor/Consultant|Gilead Sciences: Advisor/Consultant|Gilead Sciences: Grant/Research Support|Janssen: Advisor/Consultant|Janssen: Grant/Research Support|ViiV Healthcare: Advisor/Consultant|ViiV Healthcare: Grant/Research Support.

**Figure 1:**
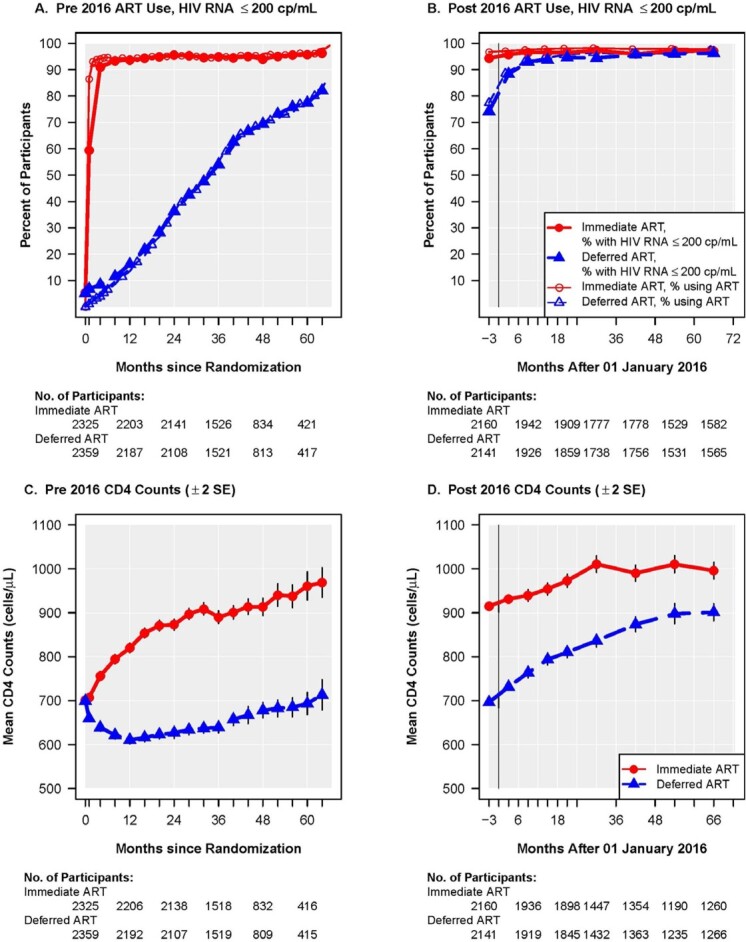
ART Use, HIV RNA ≤ 200 cp/mL, and CD4 Cell Count, Pre 2016 and Post 2016

**Figure 2:**
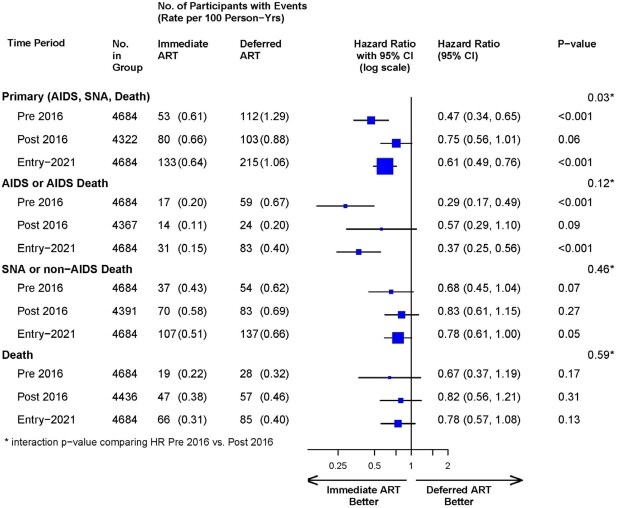
Hazard Ratios Comparing Event Rates Within Treatment Groups, by Time Period

